# A Clinical-Radiomic Model for Predicting Indocyanine Green Retention Rate at 15 Min in Patients With Hepatocellular Carcinoma

**DOI:** 10.3389/fsurg.2022.857838

**Published:** 2022-03-24

**Authors:** Ji Wu, Feng Xie, Hao Ji, Yiyang Zhang, Yi Luo, Lei Xia, Tianfei Lu, Kang He, Meng Sha, Zhigang Zheng, Junekong Yong, Xinming Li, Di Zhao, Yuting Yang, Qiang Xia, Feng Xue

**Affiliations:** ^1^Department of Liver Surgery, Renji Hospital, School of Medicine, Shanghai Jiao Tong University, Shanghai, China; ^2^Department of Instrument Science and Engineering, School of Electronic Information and Electrical Engineering, Shanghai Jiao Tong University, Shanghai, China; ^3^Department of Medical Imaging, Zhujiang Hospital, Southern Medical University, Guangzhou, China; ^4^Institute of Computing Technology, Chinese Academy of Sciences, Beijing, China

**Keywords:** indocyanine green retention rate at 15 min, radiomics, machine learning, post hepatectomy liver failure, hepatocellular carcinoma

## Abstract

**Purpose:**

The indocyanine green retention rate at 15 min (ICG-R15) is of great importance in the accurate assessment of hepatic functional reserve for safe hepatic resection. To assist clinicians to evaluate hepatic functional reserve in medical institutions that lack expensive equipment, we aimed to explore a novel approach to predict ICG-R15 based on CT images and clinical data in patients with hepatocellular carcinoma (HCC).

**Methods:**

In this retrospective study, 350 eligible patients were enrolled and randomly assigned to the training cohort (245 patients) and test cohort (105 patients). Radiomics features and clinical factors were analyzed to pick out the key variables, and based on which, we developed the random forest regression, extreme gradient boosting regression (XGBR), and artificial neural network models for predicting ICG-R15, respectively. Pearson's correlation coefficient (R) was adopted to evaluate the performance of the models.

**Results:**

We extracted 660 CT image features in total from each patient. Fourteen variables significantly associated with ICG-R15 were picked out for model development. Compared to the other two models, the XGBR achieved the best performance in predicting ICG-R15, with a mean difference of 1.59% (median, 1.53%) and an *R*-value of 0.90. Delong test result showed no significant difference in the area under the receiver operating characteristic (AUROCs) for predicting post hepatectomy liver failure between actual and estimated ICG-R15.

**Conclusion:**

The proposed approach that incorporates the optimal radiomics features and clinical factors can allow for individualized prediction of ICG-R15 value of patients with HCC, regardless of the specific equipment and detection reagent (NO. ChiCTR2100053042; URL, http://www.chictr.org.cn).

## Introduction

Hepatocellular carcinoma (HCC) is one of the common malignant tumors and accounts for more than 90% of liver cancers (1, 2). Curative hepatectomy is the preferred therapy for patients with HCC (3, 4). To date, although perioperative management and surgical techniques have greatly improved, hepatic resection is often burdened by post hepatectomy liver failure (PHLF), which is a life-threatening complication (5). The PHLF is a leading cause of mortality after surgical resection and is closely related to prolonged hospital stays, increased treatment expenses, and long-term survival (6, 7). Accurate assessment of hepatic functional reserve before the operation is of great significance in reducing the occurrence of PHLF, and this is especially true of patients with HCC with hepatic cirrhosis and impaired hepatic function (8).

Currently, although several tools have been developed for assessment of hepatic functional reserve, the indocyanine green (ICG) clearance test remains to be the most commonly used and well-established method in Eastern countries, and recent evidence reveals that this method can be also applicable to Western populations (9–11). In the clinical setting, ICG retention rate at 15 min after intravenous injection (ICG-R15) is the most frequently utilized quantitative indicator. The previous study revealed that patients with an ICG-R15 value of ≤ 10% can be tolerant of major hepatectomy (12). Wang et al. demonstrated that ICG-R15 was a more reliable predictor of hepatic functional reserve than traditional scoring systems such as the model for end-stage liver disease score and Child-Pugh grade (10). Moreover, many studies have demonstrated that the ICG-R15 acts as an important role in minimizing PHLF and postoperative mortality (13, 14). However, some patients may be allergic to ICG photosensitive dye, and more importantly, the ICG clearance test relies on expensive equipment and detection reagents, which makes it difficult for clinicians to assess hepatic functional reserve in some medical institutions that lack the equipment (15). Therefore, a simplified alternative tool was urgent to be developed to assist these clinicians to rapidly and safely obtain ICG-R15 value without the specialized equipment.

Nowadays, radiomics technology plays an increasingly central role in medical image analysis and is expected to open a broad scope of future research of medical research. The technology has been widely utilized and is becoming increasingly popular in hepatology, such as predicting microvascular invasion and tumor recurrence for HCC, using contrast-enhanced CT (16, 17). Moreover, recent studies reported that two-dimensional (2D) CT texture analysis has been applied in disease diagnosis (18, 19). Compared to 3D images, 2D regions of interest (ROIs) are easier to obtain and faster to calculate, and, thus, a 2D CT texture analysis was introduced to predict ICG-R15 in this study.

Hence, this study aims to develop a novel approach to predict ICG-R15 based on contrast-enhanced CT images and available clinical data, and, thus, provide reference evidence for clinical treatment in medical institutions that lack specialized equipment.

## Materials and Methods

### Study Cohort

Between May 2015 to October 2021, patients who underwent hepatic resection at the Department of Liver Surgery of Renji Hospital, Shanghai Jiao Tong University School of Medicine were consecutively enrolled for this study, including a subcohort with follow-up scans. The inclusion criteria of the study cohort were: (1) patients aged ≥18 years; (2) patients with postoperative pathological confirmed HCC; (3) receiving ICG clearance test within 7 days prior to surgery (4) receiving contrast-enhanced CT scanning within 30 days before the operation. The exclusion criteria were missing important clinical data and CT images of insufficient quality. The study cohort was randomly grouped into two datasets for training (70%) and testing (30%).

Before the operation, the criteria for judging patients suitable for HCC resection were: (1) no fatal cardiopulmonary or other systemic diseases, (2) BCLC 0/A (no macrovascular invasion or distant/ lymphatic metastasis), (3) Child-Pugh grade A/B, and (4) ICG-R15 < 30%.

We collected portal venous phase CT images and clinical information including demographic data, biochemical tests, and documented events of PHLF from medical records. The CT image acquisition is described in Supplementary S1.

The study has been registered in the Chinese Clinical Trial Registry center (No. ChiCTR2100053042). The institutional review board of our hospital has approved this study. Informed consent was waived given the retrospective nature of this study.

### ROI Segmentation and Feature Extraction

Two experienced radiologists (5- and 8-years' experience in abdominal imaging, respectively), unaware of clinical information, reviewed and annotated all the CT images. The 2D ROI of hepatic parenchyma was manually segmented using the 3D slicer software (Version 4.11.0). The 2D ROI delineated the largest cross-sectional area of hepatic parenchyma. Before feature extraction, images were resampled to a 1.25 mm × 1.25 mm voxel spacing to maintain the detailed in-plane information. We extracted 660 CT image features in total from each ROI using Pyradiomics (Version 3.0.1)(20). The detailed descriptions about feature extraction are shown in Supplementary S2.

### Feature Selection

Firstly, to assess the intraobserver and interobserver reproducibility of radiomics features, we randomly chose 45 CT images to calculate the intra- and inter-class correlation coefficients (ICCs). To ensure the reproducibility of the analysis, only the CT image features with both intra- and inter-observer ICC >0.75 were selected for further analysis. The 2D ROI labeling for the remaining 305 cases was completed by a radiologist with 8 years of experience. Next, we adopted the Spearman correlation coefficient (Rho) to evaluate correlations between any two CT image features. If two features are highly correlated (i.e., |Rho| > 0.9), either of the two features are excluded.

Finally, the correlation between ICG-R15 and radiomics features or clinical factors was evaluated. The |Rho| ≤ 0.3 is considered to be of no correlation and, thus, significant variables with |Rho| > 0.3 were employed to develop predictive models.

### Diagnosis and Definitions

The HCC was diagnosed based on histopathological examination of resected hepatic specimens. The extent of resection was categorized as minor (<3 Couinaud liver segments) and major hepatectomy (≥3 Couinaud liver segments) (21). According to the International Study Group of Liver Surgery definition, serum total bilirubin and international normalized ratio increased on or after postoperative day 5 was considered to be PHLF (22, 23). A PHLF grade A requires no change in the regular clinical management. A PHLF Grade B can cause a deviation from the routine course and requires essential non-invasive procedures, and PHLF grade C requires invasive therapeutic intervention. Thus, PHLF grades B and C are considered clinically significant (22).

### ICG-R15 Measurement

The ICG clearance test was carried out after 6 h of fasting. We injected ICG reagent into the patient at a standard dose of 0.5 mg/kg through a peripheral vein of the forearm. A spectrophotometry probe was utilized to monitor changes in ICG concentrations, and ICG-R15 was obtained *via* Analyzer equipment (DDG-3300K, Japan) (24).

### Model Development and Evaluation

We developed three machine learning models to predict ICG-R15 based on correlation analysis results, namely, random forest regression (RFR), extreme gradient boosting regression (XGBR), and artificial neural network (ANN) models. The best parameter combinations of models were captured by the five-fold cross-validated grid-search method (25, 26). The developed models were finally determined based on the selected variables and appropriate model parameters (Python, version 3.6.2; scikit-learn package, version 0.24). The agreement between actual and estimated ICG-R15 was evaluated using Pearson's correlation coefficient (*R*) and a confusion matrix.

### Follow-Up

After resection, patients were followed up every 3 months for the first year and twice per year thereafter. Tumor recurrence was diagnosed by serum levels of alpha-fetoprotein and medical imaging examination (ultrasonography, contrast-enhanced CT, or MRI). Disease-free survival (DFS) was regarded as the interval between the date of surgery and the date of first recurrence or last follow-up or death.

### Statistical Analysis

Data analysis was conducted using the SPSS version. 22.0 or GraphPad Prism version. 8.0. Continuous data were described by the median ± interquartile range (IQR) and compared using the Mann–Whitney U test or Student's *t*-test. The comparison of categorical variables was performed by the Chi-square test.

We performed an area under the receiver operating characteristic (AUROC) analysis to assess the performance of ICG-R15 for predicting PHLF grade B-C. Optimal cut-off values were captured to maximize the sum of sensitivity and specificity. The AUROCs for predicting PHLF between actual and estimated ICG-R15 were compared using the Delong test [MedCalc software (version.20)].

The relationship between estimated ICG-R15 and tumor recurrence was assessed using univariable Cox regression analysis. The Kaplan–Meier method was adopted to estimate disease-free survival and the comparison of curves was conducted by log-rank test. All significant tests were 2- sided and *P* < 0.05.

## Results

### Baseline Characteristics of the Patients

**Figure 1:**
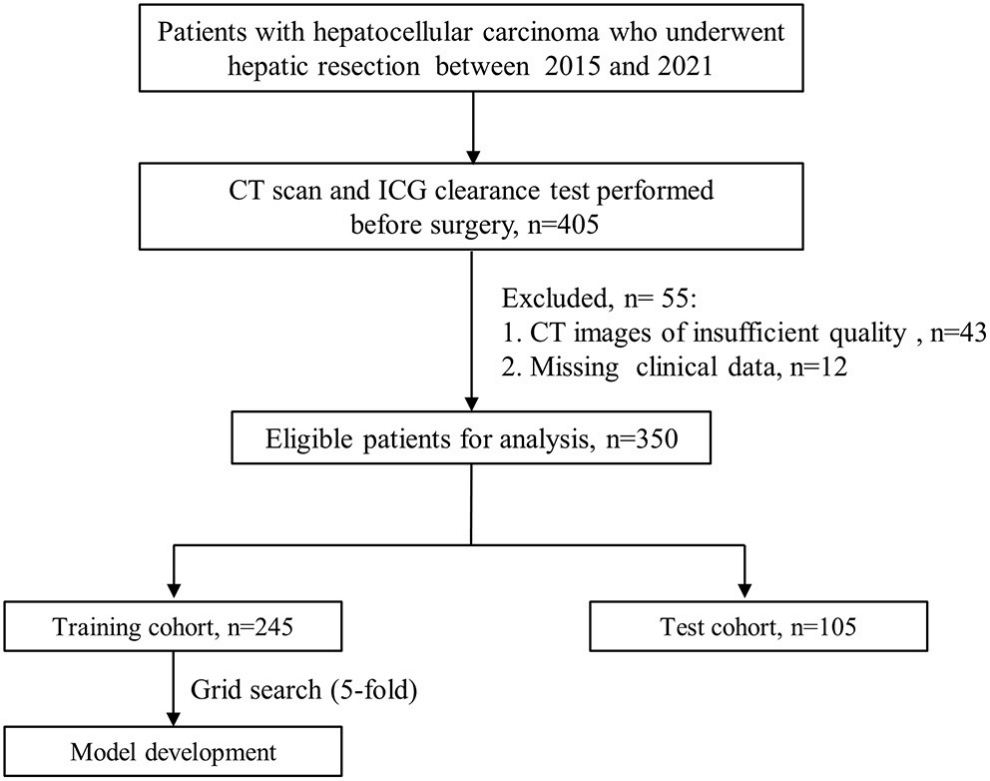
Flow diagram of model development. ICG, indocyanine green; CT, computed tomography.

Out of the 350 eligible patients, 70% (*n* = 245) patients who underwent hepatectomy were randomly included in the training cohort. The remaining 30% (*n* = 105) of patients were assigned to an independent test cohort. The flow diagram of model development was displayed in Figure 1.

The median age ± IQR of 350 patients was 58 years ± 15.8, including 282 men and 68 women. The number of PHLF grades B-C was 38 (15.5%) in the training cohort and 15 (14.3%) in the test cohort. Supplementary Table 1 showed the detailed baseline characteristics of recruited patients. Data analysis results revealed no significant difference between the two cohorts.

### Selection of Radiomics Features and Clinical Parameters

We extracted 660 CT image features in total from each patient's ROI. Based on inter-and intra-observer agreement analysis, 408 CT image features with ICC >0.75 were considered to be robust for model construction. Then, correlation analysis results indicated that 159 radiomics features and 15 clinical factors showed a significant correlation with ICG-R15, whereas only 14 variables with |Rho| > 0.3 were finally selected for model development (Table 1).

**Table 1:** Correlation analysis between indocyanine green retention rate at 15 min (ICG-R15) and variables in the training cohort.

**Variables**	**Correlation coefficient**	**P-value**
**Clinical factors**		
TBA	0.450	<0.001
DBIL	0.420	<0.001
PA	−0.403	<0.001
ALBI	0.371	<0.001
**Radiomics features**		
LoG_2mm_firstorder_Median	−0.325	<0.001
LoG_2mm_firstorder_Uniformity	0.374	<0.001
LoG_2mm_glszm_HighGrayLevelZoneEmphasis	0.336	<0.001
LoG_2mm_gldm_LargeDependenceHighGrayLevelEmphasis	−0.337	<0.001
LoG_4mm_ glcm_Imc2	−0.342	<0.001
LoG_4mm_firstorder_Entropy	−0.414	<0.001
LoG_4mm_ firstorder_90Percentile	−0.323	<0.001
LoG_4mm_ glszm_GrayLevelVariance	−0.491	<0.001
LoG_4mm_ gldm_DependenceEntropy	−0.330	<0.001
LoG_4mm_ glszm_LargeAreaLowGrayLevelEmphasis	0.315	<0.001

*Spearman correlation coefficient (Rho) was adopted to evaluate the association between ICG-R15 and radiomics features or clinical factors. Variables with |Rho| > 0.3 were picked out for model development*.

*ICG-R15, indocyanine green retention rate at 15 min; TBA, total bile acid; DBIL, direct bilirubin; PA, prealbumin; ALBI, albumin-bilirubin; LoG, Laplacian of Gaussian*.

Of note, data analysis result revealed that the strongest correlation between ICG-R15 and Laplacian of Gaussian (LoG)_4mm_glszm_GrayLevelVariance (Rho = −0.491, *p* < 0.0001) existed. The LoG_4mm_ glszm_LargeAreaLowGrayLevelEmphasis was the weakest factor related to ICG-R15 (Rho = 0.315, *p* < 0.0001).

### Development and Validation of Multiple Models

In the training cohort, the optimal parameters used in our analysis were listed in Supplementary S3. The predictive performance of models is presented in Table 2. Notably, a great agreement was observed between actual and estimated ICG-R15 (Table 2). Besides, the variable's importance of developed models excluding ANN was measured, and the top seven important variables were displayed in Supplementary Figure 1 for reference. For both models, we observed that both total bile acid (TBA) and LoG_4mm_glszm_GrayLevelVariance had a great influence on model construction. More detailed information about radiomics features is shown in Supplementary S4.

**Table 2:** Predictive performance of the models.

**Models**	**Training cohort**		**Test cohort**	
	**Correlation coefficient (*R*)**	**Mean difference% (Median)**	**Correlation coefficient (*R*)**	**Mean difference% (Median)**
RFR	0.92	1.22 (1.04)	0.89	1.73 (1.57)
XGBR	0.96	0.81 (0.56)	0.90	1.59 (1.53)
ANN	0.87	1.41 (1.10)	0.85	2.06 (1.62)

*The performance of RFR, XGBR and ANN models in predicting ICG-R15 was evaluated by using Pearson correlation coefficient (R)*.

*ICG-R15, indocyanine green retention rate at 15 min; RFR, random forest regression; XGBR, extreme gradient boosting regression; ANN, artificial neural network*.

**Figure 2:**
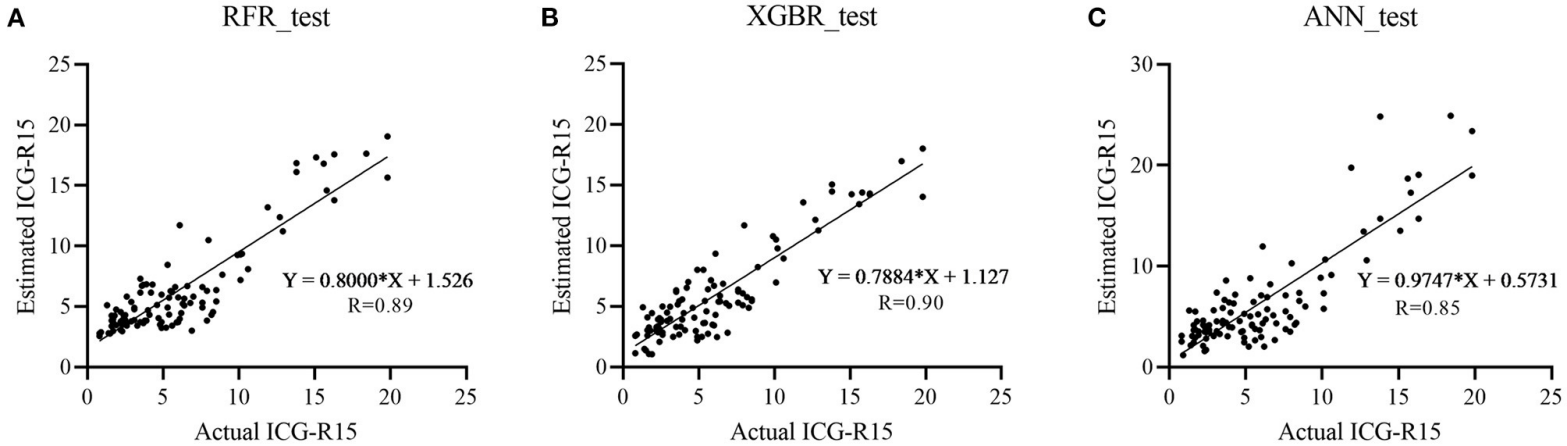
Scatter plots show actual vs. estimated ICG-R15 in the test cohort with great agreement. Three machine learning models [random forest regression (RFR), extreme gradient boosting regression (XGBR), and artificial neural network (ANN)] were developed and Pearson correlation coefficient (*R*) was adopted to assess the correlation between actual and estimated ICG-R15 in the test cohort. **(A)** RFR, *R* = 0.89; **(B)** XGBR, *R* = 0.90; **(C)** ANN, *R* = 0.85. ICG-R15, indocyanine green retention rate at 15 min; RFR, random forest regression model; XGBR, extreme gradient boosting regression model; ANN, artificial neural network model.

In the test cohort, the XGBR model showed the best predictive performance with an *R*-value of 0.90 compared to RFR (*R* = 0.89) and ANN (*R* = 0.85) models (Table 2). The scatter plots are shown in Figure 2.

Of RFR, the XBGR, and the ANN model, the overall mean difference between actual and estimated ICG-R15 was 1.73% (median, 1.57%), 1.59% (median, 1.53%), and 2.06% (median, 1.62%), respectively (Table 2).

Additionally, a confusion matrix was also calculated for the three models (Supplementary Table 2). By using the actual ICG-R15 as the reference standard, the performance of XGBR model for identifying a patient with ICG >10% was evaluated, and sensitivity and specificity were 90.91% [95% confidence intervals (CI): 83.07, 95.32%) and 100% (95% CI: 81.57, 100%)], respectively. Similarly, for RFR and ANN model, the predictive performance was 93.18% (95% CI: 85.91, 96.84%) and 100% (95% CI: 81.57, 100%), and 97.73% (95% CI: 92.09, 99.6%) and 88.24% (95% CI: 65.66, 97.91%), respectively.

As mentioned above, the XGBR model outperformed other models in the test cohort and, therefore, the XGBR model was determined for further analysis in this study. Besides, Supplementary Figure 2 presented three representative cases with hepatic cirrhosis to show the predictive performance of the model.

### Diagnostic Performance of Actual and Estimated ICG-R15 for the Distinction of PHLF Grades B-C in the Test Cohort

**Figure 3:**
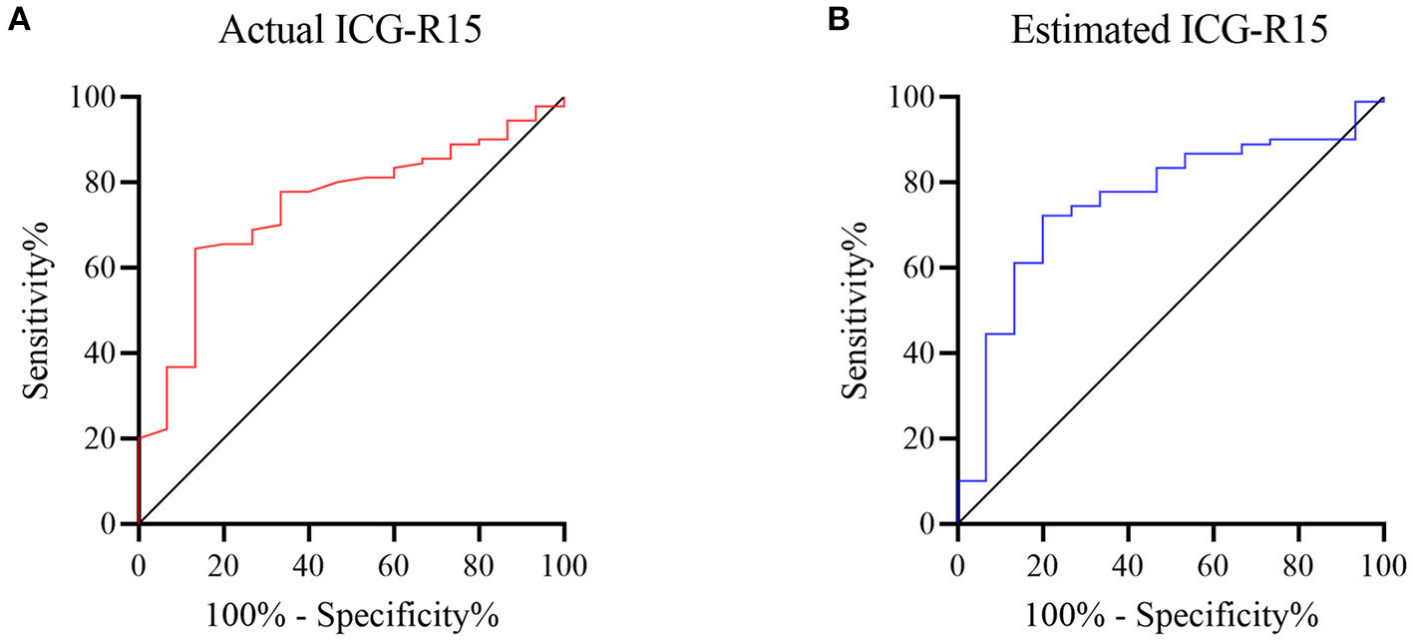
ROC curves of actual and estimated indocyanine green retention rate at 15 min [ICG-R15(XGBR)] for predicting post hepatectomy liver failure (PHLF) grades B-C. Receiver operating characteristic (ROC) analysis was used to evaluate the performance of actual and estimated ICG-R15(XGBR) for predicting PHLF grades B-C. The area under ROCs of actual **(A)** and estimated ICG-R15(XGBR) **(B)** were 0.74 and 0.75, respectively. ROC, receiver operating characteristic; ICG-R15, indocyanine green retention rate at 15 min; XGBR, extreme gradient boosting regression model; PHLF, post hepatectomy liver failure.

The diagnostic performance of actual and estimated ICG-R15 is shown in Figure 3. For predicting PHLF grades B-C, the AUROC values of actual and estimated ICG-R15 were 0.74 (95% CI: 0.62–0.86) and 0.75 (95% CI: 0.63–0.88), respectively. The cut-off value was determined to be 5.6% (64.4% sensitivity, 86.7% specificity) and 5.6% (72.2% sensitivity, 80% specificity), respectively. Notably, the Delong test result showed no significant difference in the AUROCs for predicting PHLF between actual and estimated ICG-R15 (*z*: 0.27, *P* = 0.788).

### Paired ICG Clearance Tests

**Figure 4:**
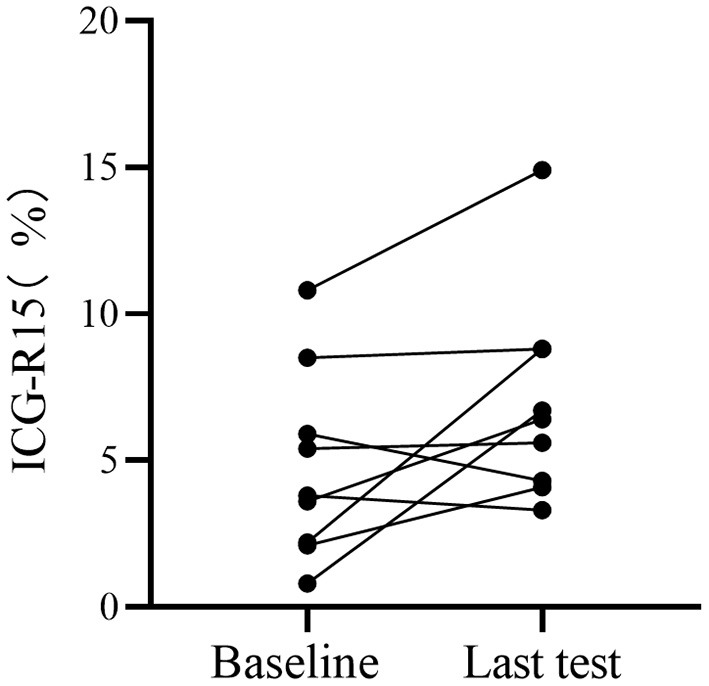
Analysis of hepatic functional reserve in paired ICG clearance tests. Differences in ICG-R15 in last examination compared with baseline. ICG-R15, indocyanine green retention rate at 15 min.

**Figure 5:**
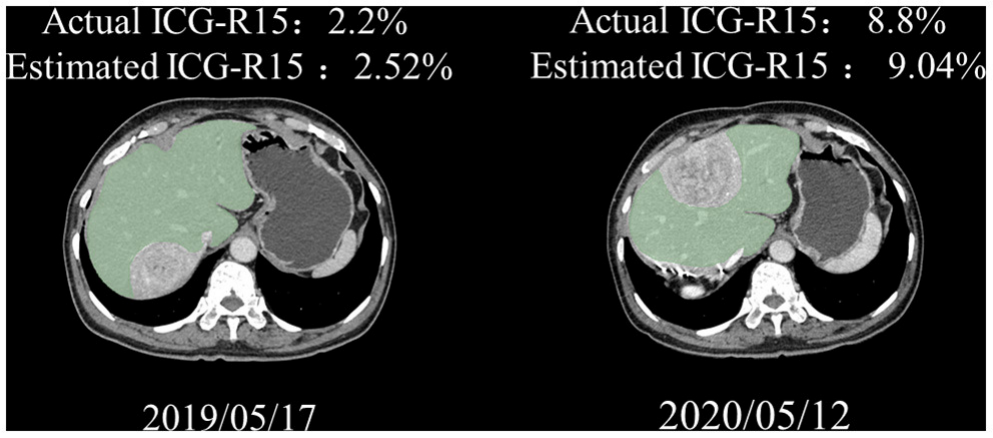
Representative paired CT images of a 68-year-old man. Great agreement was observed between actual and estimated ICG-R15. ICG-R15, indocyanine green retention rate at 15 min; CT, computed tomograph.

A subcohort of 9 patients with HCC underwent paired ICG clearance tests and contrast-enhanced CT scans, with a median time interval of 16.3 months (4–31 months) between two tests. The repeated ICG clearance test was performed for clinical reasons (i.e., tumor recurrence). The ICG-R15 value increased by an average of 2.67% between their initial and last examination, wherein 2 patients out of 9 increased in ICG-R15 value by at least 5% and the others remained within 5% (Figure 4). Additionally, compared with the first test, a total of 8 people's hepatic functional reserve decreased (Figure 5).

### The Application of Estimated ICG-R15 (XGBR Model) in Evaluating HCC Recurrence and Disease-Free Survival

**Figure 6:**
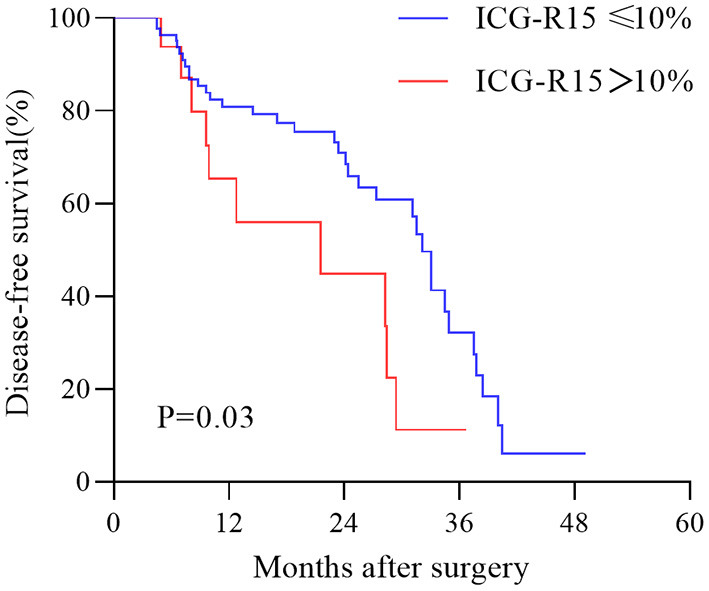
Kaplan–Meier estimation of disease-free survival in patients with hepatocellular carcinoma (HCC) according to the stratification of ICG-R15. HCC, hepatocellular carcinoma; ICG-R15, indocyanine green retention rate at 15 min.

In the test cohort, 45 patients had tumor recurrence (Supplementary Table 1). The Median time of follow-up and disease-free survival was 19.7 months (IQR, 22 months) and 15.9 months (IQR, 20.3 months), respectively. Univariable Cox regression analysis result showed that estimated ICG-R15 > 10% was significantly associated with higher HCC recurrence (Hazard ratio: 2.22, 95%CI: 1.079–4.567, *p* = 0.03). The Kaplan-Meier survival curve revealed that patients with estimated ICG-R15 > 10% before the operation had poorer DFS than those with estimated ICG-R15 ≤10% (Figure 6).

## Discussion

To our knowledge, the current retrospective study is the first report showing a novel approach for predicting ICG-R15 by combining radiomics features with clinical factors. Our study demonstrated that the XGBR model can accurately predict ICG-R15 in patients with HCC, with an *R*-value of 0.90. Delong test results showed no significant difference in the AUROCs for PHLF prediction between actual and estimated ICG-R15. The top 7 important variables of the XGBR model included four CT image features and three clinical factors [TBA, direct bilirubin (DBIL), and prealbumin (PA)].

Radiomics analysis is a useful and reproducible method and is widely used in hepatology. A recent study suggested that a computational approach was developed to improve the diagnostic capacity of the presence of microvascular invasion in HCC patients before surgery and achieve an AUROC of 0.909 (16). Mokrane et al. (27) have developed a radiomics model to differentiate tumor tissue and, thus, enhance clinicians' decision-making in HCC diagnosis, with an AUROC of 0.74. It is well documented that the proposed nomogram can accurately predict progression-free survival prior to operation (28). In the present study, the radiomics model we developed can provide a reliable method for quantitative assessment of the degree of liver damage represented by ICG-R15. The approach can assist surgeons to predict PHLF in medical institutions, which lack the specialized equipment for ICG clearance rate tests. Besides, the model may be used for estimating disease-free survival and provide a reference for comprehensive therapy in the future.

Reportedly, an ICG-R15 >10% was a significant predictive indicator of HCC recurrence (29). According to a recent study, ICG-R15 was of significance for predicting HCC recurrence (30). Besides, previous studies reported that elevated ICG-R15 was significantly linked to poor DFS (31, 32). In this study, we found estimated ICG-R15 >10% showed a significant correlation with higher HCC recurrence and disease-free survival in the estimated ICG-R15≤10% group were significantly longer than in the estimated ICG-R15 >10% group. Our result revealed that the model may be applied in evaluating prognosis for patients with HCC undergoing hepatectomy, but this still needs further confirmation in a larger population. As far as we know, hepatic cirrhosis is a key risk factor for the occurrence and recurrence of HCC (33, 34). The elevated ICG-R15 was positively correlated with a higher incidence of hepatic cirrhosis, which may be an important cause for the poor prognosis of people with higher ICG-R15 (35, 36).

In previous studies, the predictive performance of the models based solely on serological indicators was not satisfactory and could not be utilized in clinical practice (37, 38). Therefore, in this study, demographics, biochemical, and CT images were collected based on literature reports and clinical availability. Meanwhile, given the rapid development of computer-aided technology, multiple effective machine learning algorithms were also introduced to develop models. Compared with previous studies, the proposed approach in our study achieved better performance in accurately predicting ICG-R15. Also, the tool can precisely identify patients suitable for major hepatic resection (i.e., ICGR15 > 10%) and may be applied to the longitudinal assessment of hepatic functional reserve in patients with HCC.

Enhanced CT scan, a commonly performed radiological examination, can be used to clarify the nature of the lesion, determine the scope and clinical stage of the lesion, and provide the reference evidence for surgical decisions before operation. In the present study, our findings revealed that multiple individual CT image features were associated with a hepatic functional reserve and useful for predicting ICG-R15, among which, GLSZM_ GrayLevelVariance made a substantial contribution to model construction. This radiomics feature represents the variance in gray level intensities for the ROI zones (https://pyradiomics.readthedocs.io). Moreover, CT is an effective imaging modality to continuously assess hepatic functional reserve. Hobeika et al. (39) reported that the radiomic score on preoperative CT images can be applied in the assessment of hepatic functional reserve in patients with HCC. Cai et al. (40) has also proposed that changes in radiomics features of hepatic parenchyma from CT scans correlated well with hepatic functional reserve. Additionally, hepatic fibrosis is significantly linked to hepatic functional reserve (41). The CT image features can also be used to evaluate the degree of underlying hepatic fibrosis, which was closely associated with ICG-R15 (Rho = 0.49) (42, 43).

To our knowledge, a serum TBA is a sensitive biomarker for hepatic parenchymal damage and long-term survival, which can specifically reflect liver excretion function (44–46). Patients with more sufficient hepatic functional reserve had significantly more capacity for bile acid excretion (47). In our study, the analysis results revealed that ICG-R15 correlated significantly with serum TBA (Rho = 0.450). According to a previous study, the level of serum TBA correlated well with the ICG disappearance rate before surgery (48). Takahashi et al. also reported that there was a great correlation between ICG-R15 and TBA (R = 0.501) (49).

The DBIL is an important clinical indicator of hepatobiliary diseases and elevated concentrations of serum bilirubin indicate hepatocellular dysfunction (50). Our study result revealed that a positive correlation between the level of DBIL and ICG-R15 exists (Rho = 0.420). Reportedly, Furuyama et al. (51) have proposed that DBIL may reflect endogenous variations of hepatocellular more sensitively compared with total bilirubin when hepatic dysfunction occurs. The study also mentioned that DBIL may be more useful in predicting PHLF than total bilirubin. Hence, DBIL can be applied in assessing hepatic functional reserve and the association between both factors merits further research.

In addition, the serum PA synthesized by the liver is an important and sensitive indicator for the evaluation of malnutrition (52). Serum PA was found to be significantly associated with ICG-R15 (Rho = −0.403) in our study. This implied that serum PA may effectively reflect hepatic functional reserve in patients with HCC.

Our present study has several limitations. Firstly, the proposed approach remains to be further validated in a larger population. Secondly, due to patients with hepatitis B virus infection accounting for 76.9% in this study, the universality of the approach should be evaluated in patients with HCC in other etiologies such as alcoholic liver diseases. Thirdly, large-scale samples with elevated values of ICG-R15 (i.e., >10%) are necessary to obtain more robust evidence for future clinical application of this radiomics model in light of the low proportion of patients with hepatic cirrhosis in our study.

## Conclusion

This study demonstrated that the proposed approach can accurately predict ICG-R15 in patients with HCC based on easily obtained clinical factors and radiomics features. This approach may be used for longitudinal assessment of change in hepatic functional reserve regardless of the expensive equipment and adverse reactions, which is beneficial for the advancement of surgical technology in medical institutions that lack specialized equipment.

## Data Availability Statement

The original contributions presented in the study are included in the article/Supplementary Material, further inquiries can be directed to the corresponding authors.

## Ethics Statement

The studies involving human participants were reviewed and approved by Ethics Committee of Renji Hospital Affiliated to Medical College of Shanghai Jiaotong University. Written informed consent for participation was not required for this study in accordance with the national legislation and the institutional requirements.

## Author Contributions

JW, FX, HJ, YZ, YL, LX, TL, KH, MS, ZZ, JY, XL, DZ, YY, QX, and FX: study conception and design and manuscript drafting and revising. JW, HJ, YL, LX, and JY: data collection. JW, LX, TL, KH, MS, ZZ, QX, and YY: analysis and interpretation of data. XL and YL: data annotation. FX, YZ, DZ, YY, and FX: model development. All authors contributed to manuscript revision, read, and approved the submitted version.

## Funding

This study was supported by National Science and Technology major projects (No. 2018ZX10723-203).

## Conflict of Interest

The authors declare that the research was conducted in the absence of any commercial or financial relationships that could be construed as a potential conflict of interest.

## Publisher's Note

All claims expressed in this article are solely those of the authors and do not necessarily represent those of their affiliated organizations, or those of the publisher, the editors and the reviewers. Any product that may be evaluated in this article, or claim that may be made by its manufacturer, is not guaranteed or endorsed by the publisher.

## Abbreviations

HCC: hepatocellular carcinoma
PHLF: post hepatectomy liver failure
ICG: indocyanine green
ICG-R15: indocyanine green retention rate at 15 min
CT: computed tomography
2D: two dimensional
ROI: region of interest
RFR: random forest regression
XGBR: extreme gradient boosting regression
ANN: artificial neural network
IQR: interquartile range
TBA: total bile acid
LoG: Laplacian of Gaussian
AUROC: area under the receiver operating characteristic
CI: confidence interval
DBIL: direct bilirubin
PA: prealbumin.

## References

[1] Bray F Ferlay J Soerjomataram I Siegel RL Torre LA Jemal A. Global cancer statistics 2018: GLOBOCAN estimates of incidence and mortality worldwide for 36 cancers in 185 countries. CA Cancer J Clin. (2018) 68:394–424. 10.3322/caac.21492 30207593

[2] Li X Qi Z Du H Geng Z Li Z Qin S. Deep convolutional neural network for preoperative prediction of microvascular invasion and clinical outcomes in patients with HCCs. Eur Radiol. (2021). 10.1007/s00330-021-08198-w 34347160

[3] Vibert E Schwartz M Olthoff KM. Advances in resection and transplantation for hepatocellular carcinoma. J Hepatol. (2020) 72:262–76. 10.1016/j.jhep.2019.11.017 31954491

[4] Heimbach JK Kulik LM Finn RS Sirlin CB Abecassis MM Roberts LR. AASLD guidelines for the treatment of hepatocellular carcinoma. Hepatology (Baltimore, Md). (2018) 67:358–80. 10.1002/hep.29086 28130846

[5] Marasco G Alemanni LV Colecchia A Festi D Bazzoli F Mazzella G. Prognostic value of the albumin-bilirubin grade for the prediction of post-hepatectomy liver failure: a systematic review and meta-analysis. J Clin Med. (2021) 10:2011. 10.3390/jcm10092011 34066674 PMC8125808

[6] Melloul E Hübner M Scott M Snowden C Prentis J Dejong CH. Guidelines for perioperative care for liver surgery: Enhanced Recovery After Surgery (ERAS) Society Recommendations. World J Surg. (2016) 40:2425–40. 27549599 10.1007/s00268-016-3700-1

[7] Shehta A Farouk A Fouad A Aboelenin A Elghawalby AN Said R. Post-hepatectomy liver failure after hepatic resection for hepatocellular carcinoma: a single center experience. Langenbecks Arch Surg. (2021) 406:87–98. 10.1007/s00423-020-01956-2 32778915

[8] Honmyo N Kobayashi T Kuroda S Oshita A Onoe T Kohashi T. A novel model for predicting posthepatectomy liver failure based on liver function and degree of liver resection in patients with hepatocellular carcinoma. HPB (Oxford). (2021) 23:134–43. 10.1016/j.hpb.2020.05.008 32563594

[9] Hoekstra LT de Graaf W Nibourg GAA Heger M Bennink RJ Stieger B. Physiological and biochemical basis of clinical liver function tests: a review. Ann Surg. (2013) 257:27–36. 10.1097/SLA.0b013e31825d5d47 22836216

[10] Wang Y-Y Zhao X-H Ma L Ye J-Z Wu F-X Tang J. Comparison of the ability of Child-Pugh score, MELD score, and ICG-R15 to assess preoperative hepatic functional reserve in patients with hepatocellular carcinoma. J Surg Oncol. (2018) 118:440–5. 10.1002/jso.25184 30259515

[11] Kokudo T Hasegawa K Kokudo N. Assessment of preoperative liver function based on indocyanine green clearance. Hepatology. (2017) 66:675–6. 10.1002/hep.29232 28437858

[12] Ishizawa T Hasegawa K Aoki T Takahashi M Inoue Y Sano K. Neither multiple tumors nor portal hypertension are surgical contraindications for hepatocellular carcinoma. Gastroenterology. (2008) 134:1908–16. 10.1053/j.gastro.2008.02.091 18549877

[13] Søreide JA Deshpande R. Post hepatectomy liver failure (PHLF) - Recent advances in prevention and clinical management. Eur J Surg Oncol. (2021) 47:216–24. 10.1016/j.ejso.2020.09.001 32943278

[14] Fang T Long G Wang D Liu X Xiao L Mi X. A Nomogram Based on Preoperative Inflammatory Indices and ICG-R15 for Prediction of Liver Failure After Hepatectomy in HCC Patients. Front Oncol. (2021) 11:667496. 10.3389/fonc.2021.667496 34277414 PMC8283414

[15] Levesque E Martin E Dudau D Lim C Dhonneur G Azoulay D. Current use and perspective of indocyanine green clearance in liver diseases. Anaesth Crit Care Pain Med. (2016) 35:49–57. 10.1016/j.accpm.2015.06.006 26477363

[16] Xu X Zhang H-L Liu Q-P Sun S-W Zhang J Zhu F-P. Radiomic analysis of contrast-enhanced CT predicts microvascular invasion and outcome in hepatocellular carcinoma. J Hepatol. (2019) 70:1133–44. 10.1016/j.jhep.2019.02.023 30876945

[17] Ji G-W Zhu F-P Xu Q Wang K Wu M-Y Tang W-W. Radiomic Features at Contrast-enhanced CT Predict Recurrence in Early Stage Hepatocellular Carcinoma: A Multi-Institutional Study. Radiology. (2020) 294:568–79. 10.1148/radiol.2020191470 31934830

[18] Shan Q-Y Hu H-T Feng S-T Peng Z-P Chen S-L Zhou Q. CT-based peritumoral radiomics signatures to predict early recurrence in hepatocellular carcinoma after curative tumor resection or ablation. Cancer Imaging. (2019) 19:11. 10.1186/s40644-019-0197-5 30813956 PMC6391838

[19] Yang G Nie P Zhao L Guo J Xue W Yan L. 2D and 3D texture analysis to predict lymphovascular invasion in lung adenocarcinoma. Eur J Radiol. (2020) 129:109111. 10.1016/j.ejrad.2020.109111 32559593

[20] van Griethuysen JJM Fedorov A Parmar C Hosny A Aucoin N Narayan V. Computational Radiomics System to Decode the Radiographic Phenotype. Cancer Res. (2017) 77:e104–e7. 10.1158/0008-5472.CAN-17-0339 29092951 PMC5672828

[21] Walcott-Sapp S Billingsley KG. Preoperative optimization for major hepatic resection. Langenbecks Arch Surg. (2018) 403:23–35. 10.1007/s00423-017-1638-x 29150719

[22] Rahbari NN Garden OJ Padbury R Brooke-Smith M Crawford M Adam R. Posthepatectomy liver failure: a definition and grading by the International Study Group of Liver Surgery (ISGLS). Surgery. (2011) 149:713–24. 10.1016/j.surg.2010.10.001 21236455

[23] Fukushima K Fukumoto T Kuramitsu K Kido M Takebe A Tanaka M. Assessment of ISGLS definition of posthepatectomy liver failure and its effect on outcome in patients with hepatocellular carcinoma. J Gastrointest Surg. (2014) 18:729–36. 10.1007/s11605-013-2423-y 24297653

[24] Faybik P Krenn C-G Baker A Lahner D Berlakovich G Steltzer H. Comparison of invasive and noninvasive measurement of plasma disappearance rate of indocyanine green in patients undergoing liver transplantation: a prospective investigator-blinded study. Liver Transpl. (2004) 10:1060–4. 10.1002/lt.20205 15390334

[25] Morales DA Vives-Gilabert Y Gómez-Ansón B Bengoetxea E Larrañaga P Bielza C. Predicting dementia development in Parkinson's disease using Bayesian network classifiers. Psychiatry Res. (2013) 213:92–8. 10.1016/j.pscychresns.2012.06.001 23149030

[26] Ji G-W Wang K Xia Y-X Wang J-S Wang X-H Li X-C. Integrating Machine Learning and Tumor Immune Signature to Predict Oncologic Outcomes in Resected Biliary Tract Cancer. Ann Surg Oncol. (2021) 28:4018–29. 10.1245/s10434-020-09374-w 33230745

[27] Mokrane F-Z Lu L Vavasseur A Otal P Peron J-M Luk L. Radiomics machine-learning signature for diagnosis of hepatocellular carcinoma in cirrhotic patients with indeterminate liver nodules. Eur Radiol. (2020) 30:558–70. 10.1007/s00330-019-06347-w 31444598

[28] Liu F Liu D Wang K Xie X Su L Kuang M. Deep Learning Radiomics Based on Contrast-Enhanced Ultrasound Might Optimize Curative Treatments for Very-Early or Early-Stage Hepatocellular Carcinoma Patients. Liver Cancer. (2020) 9:397–413. 10.1159/000505694 32999867 PMC7506213

[29] Wu J-C Huang Y-H Chau G-Y Su C-W Lai C-R Lee P-C. Risk factors for early and late recurrence in hepatitis B-related hepatocellular carcinoma. J Hepatol. (2009) 51:890–7. 10.1016/j.jhep.2009.07.009 19747749

[30] Imura S Teraoku H Yoshikawa M Ishikawa D Yamada S Saito Y. Potential predictive factors for microvascular invasion in hepatocellular carcinoma classified within the Milan criteria. Int J Clin Oncol. (2018) 23:98–103. 10.1007/s10147-017-1189-8 28875240

[31] Hirokawa F Kubo S Nagano H Nakai T Kaibori M Hayashi M. Do patients with small solitary hepatocellular carcinomas without macroscopically vascular invasion require anatomic resection? Propensity score analysis.Surgery. (2015) 157:27–36. 10.1016/j.surg.2014.06.080 25482463

[32] Satoi S Matsui Y Kitade H Yanagimoto H Toyokawa H Yamamoto H. Long-term outcome of hepatocellular carcinoma patients who underwent liver resection using microwave tissue coagulation. HPB (Oxford). (2008) 10:289–95. 10.1080/13651820802168068 18773108 PMC2518304

[33] Roehlen N Crouchet E Baumert TF. Liver fibrosis: mechanistic concepts and therapeutic perspectives. Cells. (2020) 9. 10.3390/cells9040875 32260126 PMC7226751

[34] Conti F Buonfiglioli F Scuteri A Crespi C Bolondi L Caraceni P. Early occurrence and recurrence of hepatocellular carcinoma in HCV-related cirrhosis treated with direct-acting antivirals. J Hepatol. (2016) 65:727–33. 10.1016/j.jhep.2016.06.015 27349488

[35] Namieno T Kawata A Sato N Kondo Y Uchino J. Age-related, different clinicopathologic features of hepatocellular carcinoma patients. Ann Surg. (1995) 221:308–14. 10.1097/00000658-199503000-00014 7536406 PMC1234574

[36] Møller S la Cour Sibbesen E Madsen JL Bendtsen F. Indocyanine green retention test in cirrhosis and portal hypertension: accuracy and relation to severity of disease. J Gastroenterol Hepatol. (2019) 34:1093–9. 10.1111/jgh.14470 30221390

[37] Nanashima A Abo T Tobinaga S Nonaka T Fukuoka H Hidaka S. Prediction of indocyanine green retention rate at 15 minutes by correlated liver function parameters before hepatectomy. J Surg Res. (2011) 169:e119–e25. 10.1016/j.jss.2011.04.034 21658719

[38] Zheng J Xie W Huang Y Zhu Y Jiang L. The technique of 3D reconstruction combining with biochemistry to build an equivalent formula of indocyanine green (ICG) clearance test to assess the liver reserve function. BMC Surg. (2020) 20:283. 10.1186/s12893-020-00952-z 33183305 PMC7664104

[39] Hobeika C Cauchy F Sartoris R Beaufrère A Yoh T Vilgrain V. Relevance of liver surface nodularity for preoperative risk assessment in patients with resectable hepatocellular carcinoma. Br J Surg. (2020) 107:878–88. 10.1002/bjs.11511 32118298

[40] Cai W He B Hu M Zhang W Xiao D Yu H. A radiomics-based nomogram for the preoperative prediction of posthepatectomy liver failure in patients with hepatocellular carcinoma. Surg Oncol. (2019) 28:78–85. 10.1016/j.suronc.2018.11.013 30851917

[41] Gu J Zhang E Liang B Zhang Z Chen X Xiong M. Liver collagen contents are closely associated with the severity of cirrhosis and posthepatectomy liver failure in patients with hepatocellular carcinoma and child-pugh grade a liver function. Ann Surg Oncol. (2021) 28:4227–35. 10.1245/s10434-020-09557-5 33452603

[42] Danin PE Anty R Patouraux S Raucoules-Aimé M Gugenheim J Tran A. Non-invasive evaluation of NAFLD with indocyanine green clearance test: a preliminary study in morbidly obese patients undergoing bariatric surgery. Obes Surg. (2018) 28:735–42. 10.1007/s11695-017-2914-0 28875438

[43] Park HJ Park B Lee SS. Radiomics and Deep Learning: Hepatic Applications. Korean J Radiol. (2020) 21:387–401. 10.3348/kjr.2019.0752 32193887 PMC7082656

[44] Liu N Feng J Lv Y Liu Q Deng J Xia Y. Role of bile acids in the diagnosis and progression of liver cirrhosis: a prospective observational study. Exp Ther Med. (2019) 18:4058–66. 10.3892/etm.2019.8011 31611941 PMC6781791

[45] Manna LB Ovadia C Lövgren-Sandblom A Chambers J Begum S Seed P. Enzymatic quantification of total serum bile acids as a monitoring strategy for women with intrahepatic cholestasis of pregnancy receiving ursodeoxycholic acid treatment: a cohort study. BJOG. (2019) 126:1633–40. 10.1111/1471-0528.15926 31483939 PMC6899621

[46] Brandl K Hartmann P Jih LJ Pizzo DP Argemi J Ventura-Cots M. Dysregulation of serum bile acids and FGF19 in alcoholic hepatitis. J Hepatol. (2018) 69:396–405. 10.1016/j.jhep.2018.03.031 29654817 PMC6054564

[47] Chijiiwa K Mizuta A Ueda J Takamatsu Y Nakamura K Watanabe M. Relation of biliary bile acid output to hepatic adenosine triphosphate level and biliary indocyanine green excretion in humans. World J Surg. (2002) 26:457–61. 10.1007/s00268-001-0249-3 11910480

[48] Kurumiya Y Nagino M Nozawa K Kamiya J Uesaka K Sano T. Biliary bile acid concentration is a simple and reliable indicator for liver function after hepatobiliary resection for biliary cancer. Surgery. (2003) 133:512–20. 10.1067/msy.2003.142 12773979

[49] Takahashi H Shigefuku R Yoshida Y Ikeda H Matsunaga K Matsumoto N. Correlation between hepatic blood flow and liver function in alcoholic liver cirrhosis. World J Gastroenterol. (2014) 20:17065–74. 10.3748/wjg.v20.i45.17065 25493018 PMC4258574

[50] Tan H Liao S Pan T Zhang J Chen J. Rapid and simultaneous analysis of direct and indirect bilirubin indicators in serum through reagent-free visible-near-infrared spectroscopy combined with chemometrics. Spectrochim Acta A Mol Biomol Spectrosc. (2020) 233:118215. 10.1016/j.saa.2020.118215 32151990

[51] Furuyama T Kudo A Matsumura S Mitsunori Y Aihara A Ban D. Preoperative direct bilirubin to prothrombin time ratio index to prevent liver failure after minor hepatectomy. J Hepatobiliary Pancreat Sci. (2016) 23:763–70. 10.1002/jhbp.400 27717165

[52] Yanagaki M Haruki K Yasuda J Furukawa K Onda S Tsunematsu M. The significance of the rapid turnover protein score as a predictor of the long-term outcomes in hepatocellular carcinoma after hepatic resection. Ann Surg Oncol. (2021). 10.1245/s10434-021-10704-9 34490525

