# Supplementary material for: A Clinical-Radiomic Model for Predicting Indocyanine Green Retention Rate at 15 Min in Patients With Hepatocellular Carcinoma
Source: Front Surg. 2022 Mar 24;9:857838. doi: 10.3389/fsurg.2022.857838 (PMC8987271; doi:10.3389/fsurg.2022.857838)
Supplement: Supplementary file 1 [file Data_Sheet_1.pdf]

Supplementary Material

1 Supplementary Data

1.1 Supplementary S.1. Image acquisition

All contrast-enhanced abdominal CT images were obtained using a 64 multi–detector scanner (Discovery CT750HD, GE Healthcare, Milwaukee, USA) during breath-hold. Portal venous phase imaging was performed at 60–70 seconds after intravenous administration of nonionic iodinated contrast agent at a rate of 2–3 ml/s. All CT images were acquired at 120 kVp tube voltage and reconstructed with a 1.25-mm section thickness. The enhanced CT data was collected from Picture archiving and communication system and then saved in the format of DICOM.

1.2 Supplementary S.2. Radiomics features extraction

We extracted 660 (93*7+9) CT image features in total from each patient's image (the original image, wavelet-filter image and Laplacian of Gaussian (LoG)-filter image) using Pyradiomics (Version 3.0.1). In additional, LoG-based texture features were generated with values of 2- and 4-mm. the Wavelet transform was used for image processing using four frequency band combinations (low-low [LL], low-high [LH], high-low [HL]and high-high [HH]).

The radiomic features of images were grouped into 2 categories, including 18 first order features and texture features. Texture features are subdivided into the following classes: 24 gray level cooccurrence matrix (GLCM) features, 16 gray level size zone matrix (GLSZM) features, 16 gray level run length matrix (GLRLM) features, 14 gray level dependence matrix (GLDM) features and 5 neighborhood gray level different matrix (NGTDM) features. Besides, each original image group consists of 9 shape-based features in addition to all above features.

1.3 Supplementary S.3. Model parameters

The optimal hyperparameters used in our analysis were as follows (determine by grid search results): RFR (number of trees, 300; maximum tree depth, 5); XGBR (number of trees, 19; maximum tree depth, 7; learning rate, 0.162; subsample, 0.5); ANN (epochs, 600; hidden layers, 14; optimizer, Adam; batch size of 5; learning rate, 0.001).

1.4 Supplementary S.4. Detailed descriptions of extracted radiomics features

In total, four CT-based radiomics features were selected for the development of XGBR model, the meanings of which are described as follows:

1. GLSZM

The GLSZM features quantify gray level zones in a CT image and provide much information on the size of homogeneous zones for each grey-level. They can have small- and large-area as well as low- or high-gray emphasis.

1.1 GlSZM_GrayLevelVariance (GLSZM_GLV)

GLSZM_GLV measures the variance in gray level intensities for the ROI zones in CT images. A recent report suggested that this radiomics feature can be used for differentiating non-advanced from advanced liver fibrosis, which indicates that it may reflect the degree of liver fibrosis(1).

1.2 GlSZM_ LargeAreaLowGrayLevelEmphasis (GLSZM_LALGLE)

GLSZM_LALGLE measures the proportion in the image of the joint distribution of larger size zones with lower gray-level values. Reportedly, GLSZM_LALGLE can be applied for the classification of liver segments with low-grade vs. high-grade fibrosis. There is a positive correlation between this feature and the degree of liver fibrosis(2).

2.GLDM

GLDM features quantify gray level dependencies in a CT image.

2.1 GLDM _LargeDependenceHighGrayLevelEmphasis (GLDM_LDHGLE)

GLDM_LDHGLE measures the joint distribution of large dependence with higher gray-level values. This radiomics feature was also found to weakly correlate with liver fibrosis in a recent study(2).

3. First Order Statistics

First Order Statistics feature represent intensity distribution in the ROI based on the intensity histogram and take into account only pixel intensity, not spatial relationship of pixels.

3.1 First order _Entropy

First order _Entropy measures the amount of information in pixel values and represents the irregularity or complexity of pixel intensities in an image. It has been reported that First order _Entropy showed the significant difference between healthy and cirrhotic population(3). In a recent study, First order _Entropy was used for the development of the CT-based machine learning models for predicting the stages of liver fibrosis (4). Therefore, this radiomics feature can be used to evaluate the degree of underlying hepatic fibrosis.

Finally, more detailed information on radiomics features can be found on the website (https://pyradiomics.readthedocs.io).

References

1. Hu P, Hu X, Lin Y, Yu X, Tao X, Sun J, et al. A Combination Model of Radiomics Features and Clinical Biomarkers as a Nomogram to Differentiate Nonadvanced From Advanced Liver Fibrosis:

A Retrospective Study. *Academic radiology* (2021) 28 Suppl 1:S45-S54. doi: 10.1016/j.acra.2020.08.029.

2. Budai BK, Tóth A, Borsos P, Frank VG, Shariati S, Fejér B, et al. Three-dimensional CT texture analysis of anatomic liver segments can differentiate between low-grade and high-grade fibrosis. *BMC Med Imaging* (2020) 20(1):108. doi: 10.1186/s12880-020-00508-w.

3. Duan J, Hu C, Qiu Q, Zhang J, Meng H, Wang K, et al. Characterization of microvessels and parenchyma in in-line phase contrast imaging CT: healthy liver, cirrhosis and hepatocellular carcinoma. *Quant Imaging Med Surg* (2019) 9(6):1037-46. doi: 10.21037/qims.2019.06.12.

4. Cui E, Long W, Wu J, Li Q, Ma C, Lei Y, et al. Predicting the stages of liver fibrosis with multiphase CT radiomics based on volumetric features. *Abdom Radiol (NY)* (2021) 46(8):3866-76. doi: 10.1007/s00261-021-03051-6.

2 Supplementary Figures and Tables

2.1 Supplementary Figures

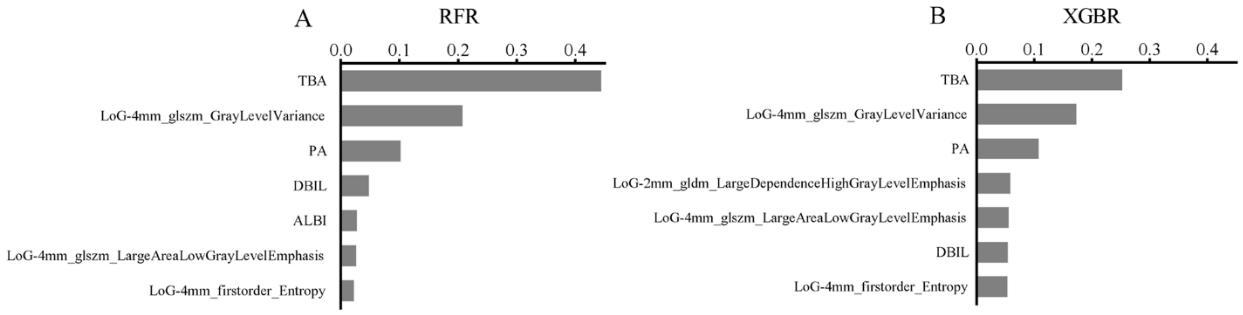

Supplementary Figure 1. Variables' importance ranking. The importance of different variables in model development was assessed. Abbreviations: RFR, random forest regression; XGBR, extreme gradient boosting regression; TBA, total bile acid; DBIL, direct bilirubin; PA, prealbumin; ALBI, albumin-bilirubin; LoG, Laplacian of Gaussian.

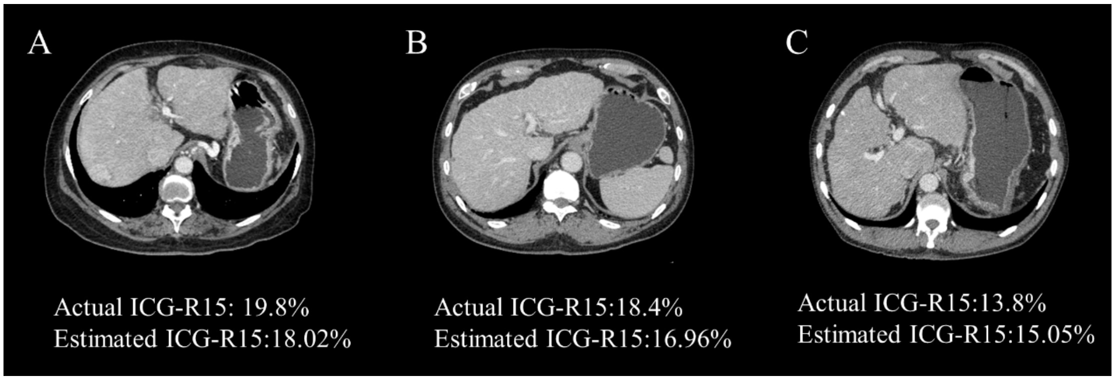

Supplementary Figure 2. Three representative cases with hepatic cirrhosis to show the predictive performance of the model. A, a 66-year-old woman; B, a 68-year-old man; C, a 48-year-old man

2.2 Supplementary Tables

Supplementary Table 1. Characteristics of patients enrolled in the study

Characteristics	Total (n=350)	Training cohort (n=245)	Test cohort (n=105)	P value
Demographics				
Age，years[a]	58(15.8)	58(14)	58(17)	0.346
Gender, males[b]	282(80.6)	202(82.4)	80(76.2)	0.175
Etiologic cause of liver disease [b]				0.180
HBV	268(76.6)	194(79.2)	74(70.5)	
HCV	15(4.3)	11(4.5)	4(3.8)	
HBV co-infected with HCV	1(0.2)	0(0)	1(0.9)	
None	66(18.9)	40(16.3)	26(24.8)	
Cirrhosis [b]				0.848
Yes	136(38.9)	96(39.2)	40(38.1)	
No	214(61.1)	149(60.8)	65(61.9)	
Coagulation function tests				
INR[a]	1.04(0.13)	1.04(0.13)	1.04(0.13)	0.895
TT (sec)[a]	18.2(2.3)	18.2(2.1)	18.1(2.4)	0.580
PT (sec)[a]	11.8(1.3)	11.8(1.4)	11.9(1.4)	0.544
PLT (10^9/L) [a]	159(91)	159(92)	159(83)	0.992

Hepatic function tests				
ICG-R15 (%) [a]	4.7(4.6)	4.6(4.0)	4.9(5.0)	0.499
ALB(g/L) [a]	41.6(6.2)	42.2(6.2)	40.9(5.5)	0.085
GGT(U/L) [a]	70(81.8)	76(83)	63(78.5)	0.294
ALT(U/L) [a]	29(23)	29(24)	29(21.2)	0.752
AST(U/L) [a]	31(22.8)	31(23)	31(22)	0.735
Cr (μmol/L)	73(17.8)	73(20.5)	70(14)	0.093
TG (mmol/L) [a]	1.1(0.70)	1.1(0.7)	1.1(0.8)	0.288
ALP (U/L) [a]	91(44.8)	91(44)	91(47)	0.665
FBG (mmol/L) [a]	5.8(2.6)	5.7(2.5)	6.2(2.6)	0.151
Urea (mmol/L) [a]	5.3(1.9)	5.3(1.9)	5.3(2.0)	0.585
Uric Acid (μmol/L) [a]	326(112.3)	328(108)	321(115)	0.482
PA (mg/L) [a]	187.4(71.8)	190.2(69.2)	183.1(75.3)	0.119
Globulin (g/L) [a]	27.6(5.9)	27.6(6.1)	27.6(5.3)	0.952
DBIL (μmol/L) [a]	4.4(2.5)	4.3(2.4)	4.5(2.3)	0.675
Total cholesterol (mmol/L) [a]	4.2(1.5)	4.2(1.5)	4.1(1.2)	0.379
TBIL (μmol/L) [a]	11.5(6.5)	11.6(6.6)	11.5(5.9)	0.962
TBA (μmol/L) [a]	7.3(8)	7.1(7.4)	7.8(10.9)	0.239
Child-Pugh score[a]	5(0)	5(0)	5(0)	0.844
MELD score[a]	7.2(1.5)	7.2(1.6)	7.1(1.4)	0.450
ALBI score[a]	-2.86(0.5)	-2.88(0.5)	-2.78(0.46)	0.082
Tumor-related parameters				
Tumor diameter (cm) [a]	4.5(5.5)	4.5(5)	4.5(4.5)	0.575
AFP (ng/ml) [a]	64.7(2026)	94.2(2246)	61.3(1082)	0.543
CEA (ng/ml) [a]	2.5(1.9)	2.5(1.9)	2.5(1.7)	0.413
CA199 (U/ml) [a]	16.8(17.4)	16.8(17.7)	17.3(20.5)	0.071
Blood testing indexes				
Hemoglobin (g/L) [a]	140(22)	141(25)	139(19)	0.542
Red cell count (10^{12}/L) [a]	4.5(0.8)	4.5(0.8)	4.6(0.7)	0.577
White cell count (10^{9}/L) [a]	5.6(2.3)	5.6(2.3)	5.4(2.3)	0.793
Lymphocyte count (10^{9}/L) [a]	25.8(12.6)	25.0(13.0)	26.2(11.6)	0.198
Percentage of monocytes (10^{9}/L) [a]	0.44(0.22)	0.44(0.22)	0.42(0.24)	0.901
Extent of surgery [b]				0.659

Minor hepatectomy	268(76.6)	186(75.9)	82(78.1)	
Major hepatectomy	82(23.4)	59(24.1)	23(21.9)	
PHLF [b]				0.770
B/C	53(15.1)	38(15.5)	15(14.3)	
A/No	297(84.9)	207(84.5)	90(85.7)	
BCLC stage [b]				0.216
0	60(17.1)	38(15.5)	22(20.9)	
A	290(82.9)	207(84.5)	83(79.1)	
No. of recurrences [b]	140(40)	95(38.8)	45(42.9)	0.475
Disease-free survival (months) [a]	15.3(18.6)	15.3(18.1)	15.9(20.3)	0.806

Note: a. Quantitative values are described as Median (IQR) and compared using the Mann–Whitney U test or Student's t test. b. Categorical variables in parentheses are percentages and compared using the chi-square test.

Abbreviations: IQR, interquartile range; HBV/HCV, hepatitis B/C virus; INR, international normalized ratio; TT, thrombin time; PT, prothrombin time; PLT, platelet count; ALB, albumin; ALT, alanine aminotransferase; GGT, γ-glutamyl transferase; AST, aspartate aminotransferase; Cr, creatinine; TG, triglyceride; ALP, alkaline phosphatase; FBG, fasting blood glucose; PA, prealbumin; DBIL, direct bilirubin; TBIL, total bilirubin; TBA, total bile acid; ICG-R15, indocyanine green retention rate at 15 min; MELD, model for end-stage liver disease; ALBI, albumin-bilirubin; AFP, α-fetoprotein; CEA, carcinoembryonic antigen; CA199, carbohydrate antigen 199; PHLF, posthepatectomy liver failure, BCLC, Barcelona Clinic Liver Cancer.

Supplementary Table 2. Confusion matrixes of models in the test cohort (n=105)

**Models**	**Actual ICG-R15**	**Estimated ICG-R15**	
		**≤10%**	**>10%**
RFR	≤10%	86	2
	>10%	4	13
XGBR	≤10%	86	2
	>10%	3	14
ANN	≤10%	86	2
	>10%	3	14

Note: By using the actual ICG-R15 as the reference standard, a confusion matrix was used for evaluate the performance of estimated ICG-R15 for identifying a patient with ICG >10%.

Abbreviations: ICG-R15, indocyanine green retention rate at 15 min; RFR, random forest regression; XGBR, extreme gradient boosting regression; ANN, artificial neural network.
